# Embryology and Morphological (Mal)Development of UPJ

**DOI:** 10.3389/fped.2020.00137

**Published:** 2020-04-07

**Authors:** Ali Avanoglu, Sibel Tiryaki

**Affiliations:** ^1^Division of Pediatric Urology, Department of Pediatric Surgery, Ege University, Izmir, Turkey; ^2^Gaziantep Maternity and Children's Hospital, Pediatric Urology, Gaziantep, Turkey

**Keywords:** ureteropelvic junction obstruction, embryology, genetics, congenital anomalies of the kidney and urinary tract, BMP4

## Abstract

Kidney parenchyma and collecting system arise from two different embryologic units as a result of a close interaction between them. Therefore, their congenital abnormalities are classified together under the same heading named CAKUT (congenital abnormalities of the kidney and urinary tract). The pathogenesis of CAKUT is thought to be multifactorial. Ureteropelvic junction obstruction (UPJO) is the most common and most investigated form of CAKUT. Despite years of experimental and clinical research, and the information gained on the embryogenesis of the kidney; its etiopathogenesis is still unclear. It involves both genetic and environmental factors. Failure in development of the renal pelvis, failure in the recanalization of ureteropelvic junction, abnormal pyeloureteral innervation, and impaired smooth muscle differentiation are the main proposed mechanisms for the occurrence of UPJO. There are also single gene mutations like AGTR2, BMP4, Id2 proposed in the etiopathogenesis of UPJO.

## Introduction

The role of embryology in medical education is often underrated. Even clinicians dealing with congenital abnormalities consider in-depth knowledge on embryology unnecessary. Studies about urinary tract obstruction date back to forties (1), but there is still scarce information on how ureteropelvic junction obstruction (UPJO) develops. We believe, clinicians and embryologists shall work together to obtain further progress. The aim of this review is to demystify the current knowledge on the embryo-pathogenesis of UPJO to clinicians to promote future research.

## The Concept Of Cakut

Congenital anomalies of the kidney and urinary tract (CAKUT) refer to all the developmental abnormalities of kidney and ureter (2). The concept of CAKUT is based on the close interaction of the ureteric bud and metanephric mesenchyme in the development of kidney and ureter.

The main steps in the formation of the metanephric kidney and ureter are; formation of the ureteric bud from the Wolffian duct, its dorsal growth into the caudal portion of the nephric cord and branching of the ureter when it invades the mesenchyme. This appositional growth continues until the formation of the terminal nephrons in 32nd week in human embryo (3).

Experimental studies with knock-out mice support this interactive development delivering the renal parenchymal and ureteric abnormalities together. In fact, most popular theory about this close interaction by Mackie and Stephens was even earlier than these. They hypothesized that the association of renal parenchymal abnormalities with vesicoureteral reflux and other ureteric abnormalities were the result of initial ectopic budding of the ureter (4).

CAKUT accounts for one of the most frequent congenital abnormalities detected by routine fetal sonography (5), but the spectrum is wide. Ureteropelvic junction obstruction is the most common form of CAKUT with an estimated incidence of 1/1,000–1,500 (6).

## Theories on UPJO Pathogenesis

The first theory was obliteration-recanalization by Ruano-Gil and Tejedo-Mateu which they raised on their findings on 45 normal human embryos of 5–55 mm (7). They said the ureter becomes obstructed beginning when the fetus is 14 mm, this process starts in the middle zone and progresses to the entire lumen, and then recanalization occurs after the fetus is 22 mm (7). Later, Alcaraz et al. also supported the existence of an obstructive phenomena of the ureter with their study on human and rat embryos (8); however, showed that this obstruction site didn't reach the ureteropelvic junction. After that, obstruction-recanalization theory to explain UPJO was abandoned by the majority. Also others think this obstruction phenomenon can only be the collapse of the ureter before the passage of the urine (2).

Other early studies about the subject were pathological analyses of the specimens with UPJO. They all noted the changes in the ureteropelvic junction (UPJ) without attribution to the etiology (9). Zhang et al. were also researchers who analyzed UPJO specimens. They showed that UPJs were thicker with enlarged muscularis propria, increased perifascicular fibrosis and inflammation in cases with intrinsic UPJO (10). They also couldn't make a statement whether these changes were causative but showed that they were not apparent in the extrinsic cases.

Miyazaki et al. showed angiotension type 1 lacking mice failed to develop a renal pelvis (11). They also showed hypoplastic smooth muscle and lacking peristalsis in the ureters of mutant mice. Reminding the results of Miyazaki's experimental study, Kajbafzadeh et al. showed increased smooth muscle cell apoptosis and collagen fibers while a decreased number of nerve terminals in the UPJO specimens compared to normal ureteropelvic junctions from autopsies (12). These studies strongly suggest defective muscle and nerve structure in the site of obstruction, but it is still unknown if these are the causative changes or the results of the obstruction. Later, Yiee et al. compared intrinsic, and extrinsic cases focusing on the muscle distribution. Their findings support a causative role by revealing a different muscle density between them (13).

Chang et al. generated an animal model of UPJO with a mutation in a calcineurin protein subunit (14). The mutant mice had abnormal renal mesenchyme and lack of a funnel-shaped ureteropelvic junction. They showed no abnormality in the nerve distribution. They correlated abnormal shape of the pelvis and faulty mesenchyme with abnormal pyeloureteral peristalsis which they concluded as the cause of UPJO.

Based on the studies about peristalsis, Lye et al. speculated that peristalsis in the urinary tract becomes more important in late gestation when the fetus stays upside down and urine travels against gravity. They concluded that failure of peristalsis results in a functional obstruction manifested by hydronephrosis (15).

In fact, none of the above studies describe the macroscopic findings of the surgeon which are as follows: mostly there is narrow but patent lumen, ureter inserts the pelvis in a level higher than ureter and pelvis first meet, they are attached to each other between these two levels and there is fibrotic tissue around. Stephen Koff has an interesting idea about this that he never published. He believes UPJO is a consequence of temporary vesicoureteral reflux during the fetal life. He says reflux disrupts the position of the ureter and UPJ, and then pelvic drainage. When this lasts long enough, it results in inflammation and the fibrotic attachments around and UPJO becomes permanent (Koff, personal communication).

Despite the above theories and two very interesting speculations, further studies are still required to reveal etiopathogenesis of UPJO.

## The Genetics

CAKUT is thought to be multifactorial. There are familial cases with different occurrence, so genetic penetrance is regarded to be incomplete or variable. Also, there are several single gene mutations like Id2, PAX2, EYA, AGTR2, BMP4, SOX17, CHD1L, DSTYK proposed by the experimental and clinical studies about the etiopathogenesis of UPJO (16–19). However, mutations in these mostly results in more than one form of CAKUT. For example, mutant mice has a 3% chance of developing CAKUT when AGTR2 is inactivated, but it can be any type and happens randomly within the same pedigree (2).

Among these, Adamts1 and Id2 are reported to lead to a more restricted phenotype resembling human UPJO (17). Interestingly, the macroscopic morphology of the kidney of the Id2 knock-out mice even shows the high-insertion of the ureter into the pelvis (17).

BMP4 also has noteworthy features. It has an essential role in embryonic development shown by the fatality of the homozygous null mutations. Heterozygous mutation results, on the other hand, in multiple abnormalities including all types of CAKUT. It is also shown to cause ectopic budding of the ureter (like Mackie and Stephens described) (20). BMP4's role may seem too wide to explain UPJO alone; however, two screening studies showed its association with UPJO (21, 22). The study from China revealed BMP4 mutation in three cases with UPJO which were not apparent in the controls (21). Same study failed to show any specific mutation in Id2 gene. The other one from Brazil showed the association of BMP4 mutation with UPJO and multicystic dysplastic kidney (22).

Despite promising results of these papers, data to acknowledge a causative role of any gene is still lacking.

## Conclusion

The etiopathogenesis and impacts of ureteropelvic junction obstruction has long been an interesting area for researchers. Despite years of clinical and experimental research, there is no solid theory or genetic mutation to explain this frequent abnormality yet.

## Author Contributions

AA and ST contributed to the literature search and drafting the manuscript of this review.

### Conflict of Interest

The authors declare that the research was conducted in the absence of any commercial or financial relationships that could be construed as a potential conflict of interest.
